# Beyond dichotomies in species and speciation

**DOI:** 10.1093/nsr/nwad018

**Published:** 2023-02-07

**Authors:** Jeffrey L Feder, Patrik Nosil

**Affiliations:** Department of Biological Sciences, University of Notre Dame, USA; Centre d'Ecologie Fonctionnelle et Evolutive, University Montpellier, CNRS, EPHE, IRD, France

Speciation is not as mysterious as Darwin once described it [1]. Nevertheless, important questions remain. As discussed by Wu [2] and highlighted in a series of papers in *National Science Review* (*NSR*), these issues equate with imposing dichotomous perspectives on speciation, when the nature of species and the processes creating them can complicate discrete categorization.

The question of what constitutes a species has a long, contentious history [3,4]. Inherent barriers to gene flow can generate increasing phenotypic, genetic and reproductive gaps between populations with time. However, defining when these gaps are sufficient to recognize ‘distinct’ species—rather than partly separated ecotypes or races—may be subjective unless reproductive isolation (RI) is complete. Consequently, Wu [2] notes, researchers delineate species differently in relation to their objectives. Thus, one may consider species as an artificial taxonomic category not different from a genus, as Darwin [1] did, despite viewing the gaps between species as real.

With respect to delineating species, Wu [2] uses the analogy of changing seasons. Earlier, Walsh [5] compared the problem to the transition from childhood to adulthood. In the analogies of Wu and Walsh, however, non-arbitrary demarcations can be made in terms of changes in day length and sexual maturity, respectively. The issue then is: does speciation usually proceed in ‘fits and bursts’, transitioning between identifiable phases recognizable as populations versus species or gradually, producing a continuum of forms not easily or universally categorized [6–8]?

The answer may sometimes be ‘yes’ and other times ‘no’ (e.g. see work on *Heliconius* butterflies [9]; and on mangroves [10], glacial fish [11], *Rhododendron* plants [12] and marine diversity [13] in this issue of *NSR*). In one of our study systems, *Timema* stick insects, there is a clear break in genome-wide differentiation such that only modest (*F*_ST_ < 0.30) and high (*F*_ST_ > 0.70) levels of differentiation occur in sympatry, interpretable as populations versus species [14]. Notably, this ‘gap’ is not seen in allopatry, where differentiation between populations gradually blends into that between species, demonstrating the context-dependent nature of divergence. In another of our study systems, *Rhagoletis* fruit flies, a break in differentiation in sympatry is not obvious [15]. For *Rhagoletis*, where speciation is often initiated by divergent ecological selection associated with host plant shifts, species may represent host races writ large [16]. This does not mean, however, that species cannot still be statistically defined, for instance, by the degree to which they form distinct genotypic clusters or can be correctly assigned to one or another host taxon [4,17,18]. The challenge then is equating these probabilistic definitions of species with the process of speciation. Groups displaying distinct gaps compared to those that do not may reflect important differences in their natural histories (e.g. the role of ecology), population genetics (e.g. the strength of selection and effect sizes of mutations versus migration and recombination rates), and biogeography (e.g. the geographic context of divergence and how it changes, effecting gene flow and standing variation through time) that are responsible for these patterns and seminal to understanding the speciation process. Thus, the use of any given species definitions may be somewhat subjective and be more representative of one phase of divergence than others [19]. However, this does not mean that these definitions do not embody some underlying biological reality with respect to process and thus can be useful for understanding species formation.

In *NSR*, Orr *et al.* [20] offer suggestions to attempt to standardize the taxonomic problem of species. While they do not provide criteria for objectively defining species, they propose a checklist of data to collect in order to help unify the delineation of taxa. The utility of this endeavor is that any researcher armed with this holistic data set could, in principle, reach the same conclusion regarding species status for a given specific definition. Thus, if phases of speciation can be equated with different species definitions [6–8,19], then this could aid greatly in better understanding the relationship of pattern and process in speciation.

Defining species could be described as an attempt to dichotomize patterns of divergence into distinguishable and named categories of biodiversity. With respect to process, speciation has also been dichotomized into categories based on underlying causal factors. A short list includes:

- Allopatric versus sympatric. Does speciation require the complete geographic isolation of populations or can it occur in sympatry or parapatry with gene flow [3]?
- Few versus many genes. Is speciation due to genes having large effect sizes on RI or is it polygenic, involving several different (likely coupled) barriers to gene flow [21–23]?
- Standing variation versus new mutations. Is speciation fueled in critical formative stages by standing genetic variation or is it the result of new mutations [24]?
- Ecological versus mutation-order. Is speciation due to divergent ecological adaptation or incompatible mutations differentially accumulating in allopatric populations [25]?

Initially framing these questions about speciation as dichotomies was important for assessing what was possible and evaluating the relative likelihoods that different factors contribute to divergence and in what chronological order. Less studied, but of increasing attention, is how these factors may interact to facilitate divergence. The papers in this issue of *NSR* highlight these interactions, perhaps best exemplified by adaptive radiation.

Adaptive radiation refers to an evolutionary pattern in which an ancestral population differentiates into an array of daughter species inhabiting a variety of different environments, with descendants differing in the traits they use to exploit those environments [26]. Adaptive radiations are important because they account for a significant portion of biodiversity [26]. There are two general hypotheses for adaptive radiation. Under the ecological hypothesis, divergent selection causes populations to differentially adapt to varying biotic or abiotic conditions [26]. These adaptations cause ecological RI between populations, contributing to speciation and, when it occurs repeatedly, producing the pattern of adaptive radiation. Under the non-ecological hypothesis [27], taxa first form by a mutation-order process due to the evolution of genomic incompatibilities in allopatry. When species come back into secondary contact, competition for shared and limited resources causes ecological character displacement [28], allowing them to coexist. Critically, the order of events—divergent selection during or after speciation—is reversed between the two hypotheses.

Examples of both ecological and non-ecological speciation exist [3]. A key question, however, is rather than alternatives, whether ecology and geography often interact to account for why some radiations and groups are more diverse than others? Such a view is part of the oscillation hypothesis [29]. Here, host-plant shifts for phytophagous insects initiate ecological speciation. But host shifts also allow for range expansions into new areas, thus creating new opportunities for allopatric speciation. Expansions into new areas can also open opportunities for shifts to novel plants, generating new chances for ecological speciation or character displacement. Taxa having the capacity for such a cyclical interplay between ecology and geography may thus be more diverse than those restricted to speciate in allopatry. Populations with a dynamic history of ecological and geographic range expansions and isolation may also harbor increased standing adaptive variation, helping perpetuate their radiation [29–32], as expressed in the mixing-isolation-mixing (MIM) model of speciation proposed for mangroves [10,33].

Finally, there is the question of what constitutes sympatry when populations may be isolated on micro-geographic scales. For example, it has been suggested that for speciation to be considered sympatric, populations must initially be panmictic, with the migration rate (*m*) = 0.5 [34]; under this definition speciation will almost never be sympatric (e.g. *m =* 0.49 would be non-sympatric), defining away the classic problem. Mayr [35] was clear that for sympatry, individuals in diverging populations must be in ‘cruising range’ of one another. Thus, migration can still be partly physically impeded between interspersed habitats for speciation to be sympatric, provided gene flow is not eliminated, and habitat choice and performance are partly genetically based [36].

In this issue of *NSR*, Sun *et al.* [11] tackle the thorny issue of sympatry versus micro-allopatry or parapatry in a study of two species of cypinid fishes in a small glacier lake in the Qinghai-Tibet Plateau. The authors conclude that the fish are not strictly sympatric, as they reside in alternate habitats that are not completely overlapping. However, more extensive details of the biology of the fishes, including movement patterns and precise quantification of times and places of mating, are needed to make a strong case for any particular mode of speciation. Similarly, Sun *et al.* [11] argue that the observation of many relatively large genomic segments between the fish that are more divergent than the rest of the genome runs counter to expectations for sympatric speciation. Although this could be true based on expectations of simple models, it may not be true if complex structural genomic variation is considered. Larger regions of differentiation, often associated with inversions or other structural elements, have empirically been observed in several cases of ecological and non-ecological divergence [3,30,32,37–41]. Theory has also shown that inversions or regions of low recombination can accumulate increasing numbers of variants adapted to alternate habitats, creating co-adapted complexes [42]. Again, a dynamic biogeographic history may facilitate this process [30,43]. In addition, in polygenic speciation, populations can rapidly transition from a phase of low divergence to a phase of high differentiation [21]. Thus, observing ‘continents’ or archipelagoes rather than small, widely dispersed islands of divergence between populations does not discount sympatry [44].

In conclusion, the take-home message for the glacial lake fish is therefore not that Sun *et al.* [11] are ‘right’ or ‘wrong’, but rather that many context-dependent patterns and processes need to be considered to understand speciation. Regardless of sympatry or micro-parapatry, the seminal point of their integrative study is that the glacial lake fish may have diverged ecologically with gene flow. In this broader context, there are many ways that different combinations of factors and processes may generate similar patterns of genomic differentiation observed between taxa. Moreover, these factors may interact in ways that go beyond describing speciation as occurring by just one way or another, being due, instead, to a combination of effects varying through time and across space. This complexity can have predictable origins based on the biology of the system involved. The collection of papers in the current issue of *NSR* highlights this perspective of moving beyond dichotomies to help solve new intricate mystery plots concerning the origins and nature of species and thus better connect pattern and process to understand the origins of biodiversity.
